# Intercellular Nanotube Mitochondrial Transplantation Strategy Mediating T Cell “Supercharging”

**DOI:** 10.34133/research.0927

**Published:** 2025-09-30

**Authors:** Baikun Liu, Jie Chen, Wenguo Cui

**Affiliations:** Department of Orthopaedics, Shanghai Key Laboratory for Prevention and Treatment of Bone and Joint Diseases, Shanghai Institute of Traumatology and Orthopaedics, Ruijin Hospital, Shanghai Jiao Tong University School of Medicine, Shanghai 200025, P. R. China.

## Abstract

The functional exhaustion of T cells in the tumor immune microenvironment is closely related to mitochondrial dysfunction. Current mitochondrial-targeted strategies have failed to restore the mitochondrial impaired function effectively. Mitochondrial transplantation technology has brought a revolution to the treatment of organelle-related diseases. Here, we first summarize the therapeutic potential and available platforms for mitochondrial transplantation, and focus on a type of mitochondrial transplantation technology mediated by tunneling nanotubes. This technology transfers functional mitochondria from bone marrow mesenchymal stem cells to CD8^+^ T cells, obtaining “supercharged T cells”, which markedly enhance the metabolic adaptability and antitumor efficacy of T cells. It provides new ideas and technical platforms for the application of organelle medicine in tumor immunotherapy.

## T Cell Exhaustion and Mitochondrial Targeting Strategies in Tumor Immunity

Tumor immunotherapy activates and enhances the patient’s own immune system to identify and eliminate tumor cells, offering precise and durable effects that are difficult to achieve with traditional treatments such as surgery, radiation, and chemotherapy. Among these therapies, T cell-based adoptive immunotherapy plays a crucial role. The antitumor efficacy and metabolic adaptability of T cells are key determinants of the success of tumor immunotherapy. Numerous studies have demonstrated that a higher level of T cell infiltration correlates positively with a good prognosis in patients with various cancers, such as breast, lung, colorectal, and melanoma [1]. However, within the tumor immune microenvironment (TIME), T cells often suffer functional impairment due to hypoxia and metabolic stress, such as the Warburg effect, eventually leading to exhaustion and a marked weakening of their antitumor capabilities [2]. T cell exhaustion in tumor immunity is closely related to mitochondrial dysfunction. As the core of cellular energy metabolism, mitochondria possess unique functions in regulating oxidative stress, autophagy, and dynamic balance. Their adaptive dynamics profoundly influence the survival and antitumor efficacy of T cells [1,2]. Declines in mitochondrial quality and adaptability are hallmarks of exhausted tumor-infiltrating lymphocytes (TILs) in hematologic malignancies and certain solid tumors [3]. To address this issue, researchers have proposed a variety of mitochondrial-targeted therapeutic strategies aimed at restoring T cell function and enhancing antitumor immunity. For example, activation of inositol-requiring enzyme 1α (IRE1α) in the unfolded protein response can regulate the expression of major histocompatibility complex class I (MHC-I) antigens in cancer cells, enhancing their immunogenicity in both mouse models and patients with malignant tumors. Research indicates that the mitochondrial fission inhibitor Mdivi-1 can suppress the up-regulation of MHC-I in cancer cells and markedly enhance the efficacy of adoptive T cell therapy in patient-derived tumor models [4]. Other strategies include early selection of T cell subsets with healthier mitochondria, genetic engineering to repair mitochondrial function, and the use of antioxidants to reduce oxidative stress. However, current methods struggle to fully restore the intact biological functions of damaged or missing mitochondria [5], and are unable to reverse the ultimate fate of the damaged T cells.

Mitochondrial transplantation has recently emerged as a transformative approach for treating organelle-related diseases. By delivering healthy mitochondria into damaged cells, this strategy can replace or repair impaired mitochondria, restoring key processes such as respiration, adenosine triphosphate (ATP) production, electron transport, and oxidative phosphorylation [6]. Baldwin et al. [7] recently reported in *Cell* a new strategy for mitochondrial transplantation via intercellular tunneling nanotubes (TNTs), transferring functional mitochondria from bone marrow mesenchymal stem cells (BMSCs) into CD8^+^ T cells. This process “supercharges” exhausted T cells, markedly enhancing their metabolic adaptability and antitumor capabilities, and offers a promising new direction for immunotherapy.

## Potential Applications and Technology Platforms for Mitochondrial Transplantation

Baldwin et al. [7] first introduced the concept of organelle medicine. Organelles are micro-organs within cells that have specific morphological structures and functions, also known as pseudo-organs or substructures. Unlike whole-organ or cell transplantation, organelle transplantation can selectively restore specific cellular processes, such as energy metabolism or protein synthesis, while avoiding the complexity of transferring entire cells and reducing the risk of immune rejection [8]. Currently, organelle transplantation primarily focuses on mitochondrial transplantation for functional tissue repair, and its potential applications have been validated in various pathological models. For example, in studies of ischemic heart disease (IHD), mitochondrial transplantation restores the energy metabolism function of the heart by providing intact respiratory chain proteins and DNA sequences that fuse with the mitochondria in damaged myocardial cells [6]. Moreover, in other disease models such as acute respiratory distress syndrome (ARDS), Parkinson’s disease (PD), spinal cord injury (SCI), and acute kidney injury (AKI), the mitochondrial transplantation is equally effective [9].

**Fig. 1.**
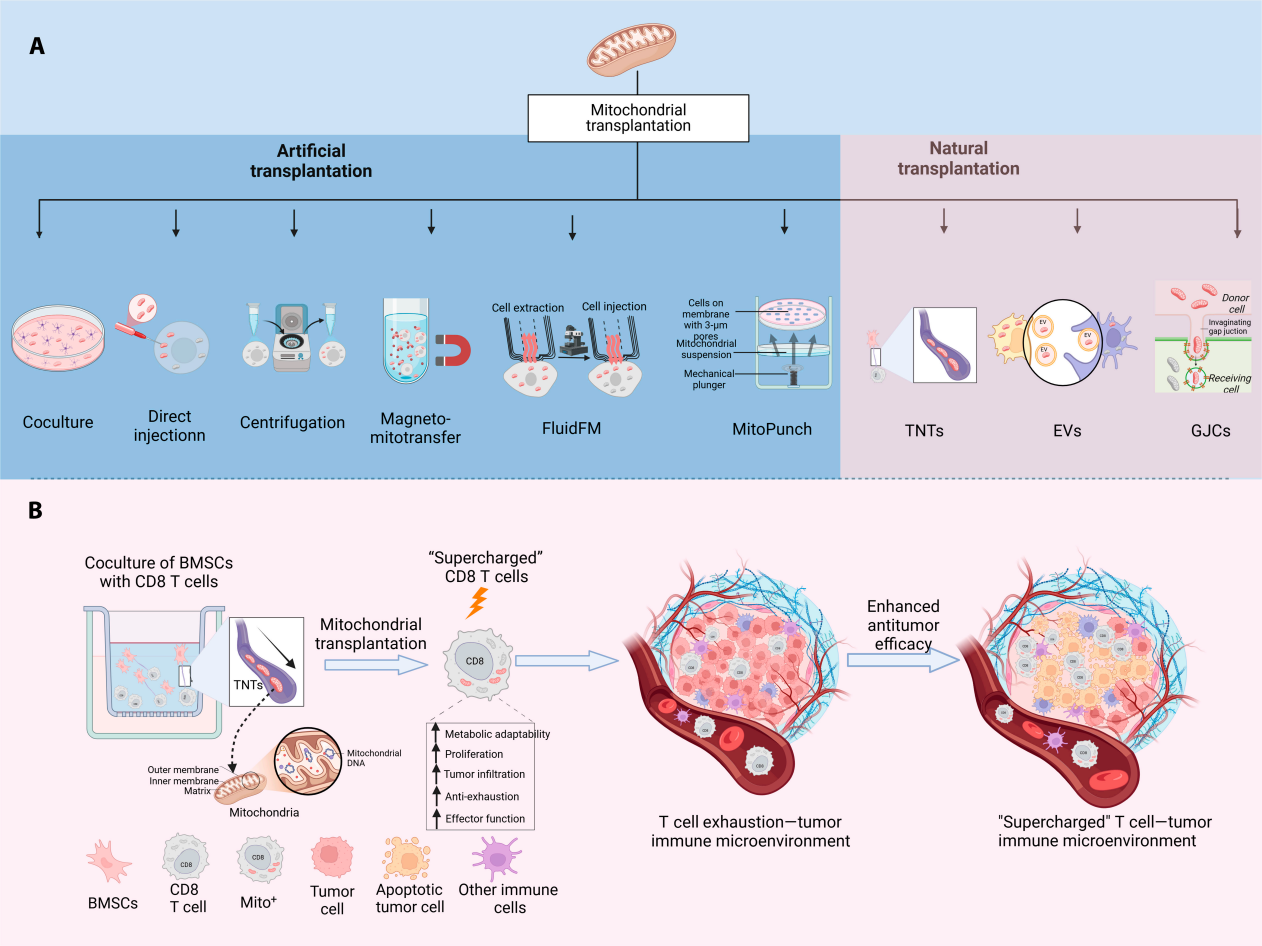
(A) Mitochondrial transplantation technology platforms. (B) TNT-mediated mitochondrial translocation enhances metabolic capacity and antitumor efficacy of T cells. Created in BioRender.

Mitochondrial transplantation can be classified as artificial or natural. In the artificial approach, mitochondria are isolated from healthy tissues and delivered into damaged sites through regional or systemic injection to supplement or replace dysfunctional networks. Beyond traditional methods such as direct injection, co-incubation, centrifugation, and magnetic delivery, platforms for mitochondrial purification and transplantation are now steadily advancing. For example, FluidFM technology combines the high-precision mechanical modulation of atomic force microscopy (AFM) with a nanoscale microfluidic system under optical detection to realize the mechanical and quantitative volume control associated with single-cell manipulation. The technique is capable of extracting, injecting, and transplanting organelles from living cells at subcellular spatial resolution, successfully realizing mitochondrial transplantation between living cells [10]. However, its limited throughput remains a major barrier to large-scale application. Therefore, researchers developed MitoPunch, a high-throughput mitochondrial transfer platform based on a photothermal nanoblade. The platform delivers mitochondria into primary or immortalized mitochondria-deficient cells by applying a pressure gradient to suspension of mitochondria isolated from mouse or human cells, and generates mitochondria with specific mitochondrial DNA (mtDNA) and nuclear DNA combinations, enabling rapid and large-scale mitochondrial transplantation [11]. However, artificial transplantation methods typically use physical or chemical means to deliver mitochondria through cell membranes, inevitably damaging mitochondrial and cellular functions and requiring high manipulation techniques. Interestingly, cells also possess natural mechanisms for mitochondrial transfer that synchronize energy across populations. These include intercellular structures such as TNTs, secreted entities like vesicles, gap junctions (GJCs), and even the release and uptake of free mitochondria [12]. The mitochondrial transfer platform reported by Baldwin et al. [7], which enhances the antitumor capabilities of T cells, is based on this natural communication mechanism between cells (Fig. 1A)

## Intercellular Nanotube-Mediated Mitochondrial Transplantation “Supercharges” T Cells

**Fig. 2.**
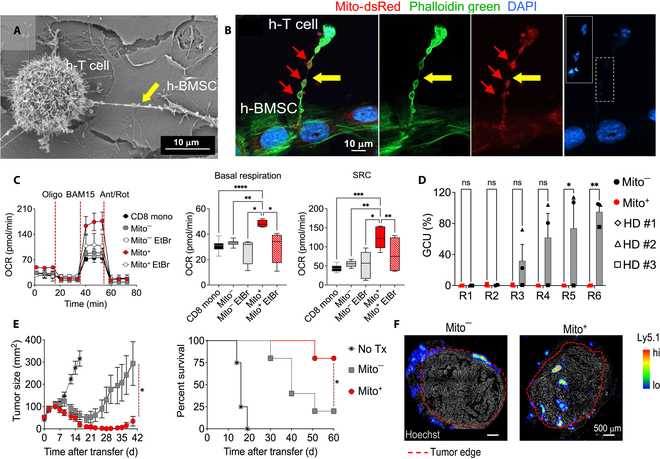
Intercellular nanotube-mediated mitochondrial transfer enhances T cell metabolic adaptability and antitumor efficacy. (A) Field-emission scanning electron microscopy images show nanotubes (yellow arrows) between BMSCs and CD8^+^ T cells in human cocultures. (B) Complete transport of mitochondria from human BMSCs to CD8^+^ T cells. (C) The oxygen consumption, basal respiration, and spare respiratory capacity of mouse CD8^+^ T cells increased after mitochondrial transplantation (Mito^+^ cells). (D) Evaluate the ability of Mito^+^ cells to kill NALM6-GL leukemia cells after repeated attacks. Mito^+^ cells maintained strong cytotoxic activity against cancer cells throughout 6 rounds of stimulation, while Mito^−^ cells began to lose their killing ability after the third round and almost completely lost function by the sixth round. (E) Mito^+^ cells mediated a more robust tumor regression compared with Mito^−^ cells, significantly prolonging mouse survival. (F) Mitochondrial transfer enhances the anti-solid tumor immunity of CD8^+^ T cells, reducing the tumor size. Reproduced with permission [7]. Copyright 2024, Cell Press.

Intercellular TNTs are F-actin-based cellular protrusions that mediate the transfer of membranes, cytoplasmic components, and organelles for long-distance intercellular communication connections [12]. Interestingly, back in November 2021, the team discovered the presence of TNTs between tumor cells and T cells and confirmed that tumor cells steal mitochondria from T cells via TNTs, thereby achieving immune escape. Moreover, inhibiting the assembly mechanism of TNTs markedly reduced mitochondrial transfer and prevented immune cell exhaustion [13]. Based on these findings, Baldwin et al. [7] describe an innovative platform for delivering exogenous mitochondria to T cells via TNTs, as described in a *Cell* study (Fig. 1B). This platform employs BMSCs to transfer mitochondria to CD8^+^ T cells through endogenous intercellular communication to achieve long-term implantation of donor mitochondria, thereby obtaining “supercharged” T cells to enhance their antitumor capabilities (Fig. 2A and B). When cocultured with BMSCs, CD8^+^ T cells with donor mitochondria (Mito^+^) exhibited higher basal and residual respiratory capacities and markedly improved metabolic adaptations compared to CD8^+^ T cells without donor mitochondria (Mito^−^) (Fig. 2C). Functional analyses and proteomics and transcriptomics studies revealed that Mito^+^ cells have important advantages in cell expansion, survival, tumor penetration, resistance to exhaustion, and differentiation into highly functional killer cells (Fig. 2E and F). For example, mitochondrial transfer conferred anti-fatigue ability to CD19-speciﬁc chimeric antigen receptor (CAR) T cells, and Mito^+^ cells consistently maintained potent cytotoxic activity in 6 rounds of repeated stimulation experiments on NALM6-GL leukemia cells, whereas Mito cells progressively lost their killing ability from the third round to the sixth round of almost complete exhaustion (Fig. 2D). Furthermore, researchers confirmed that mitochondrial transfer between BMSCs and T cells depends on TLN2, providing an important regulatory target for future mitochondrial intercellular transfer.

Of course, mitochondrial transfer for intercellular communication exists between a variety of cells. For example, mesenchymal stromal cells (MSCs) can support endothelial cell (EC) engraftment into ischemic tissues and promote the formation of functional blood vessels through mitochondrial transfer. Interestingly, this effect is transient: Exogenous mitochondria in ECs rapidly undergo mitophagy, leading to a “regression” of mitochondrial content and ATP production. This “regression” is critical for EC implantation, as knockdown of key components mediating mitochondrial autophagy with short hairpin RNAs inhibits successful EC implantation. Remarkably, even transfection of activity-deficient mitochondria (depolarized or mtDNA-less mitochondria) exerted the same effect, and exogenous mitochondria did not integrate with the mitochondrial pool of endogenous ECs [14]. However, in the study by Baldwin et al. [7], mitochondrial transfer can markedly enhance the mitochondrial quality of CD8^+^ T cells in the long term, maintaining cellular durability and the heritability of cell division. In both physiological homeostasis and the pathological context of cancer, transplanted mitochondria promoted CD8^+^ T cell proliferation and function without triggering mitophagy—even under extreme conditions such as hypoxia, nutrient deprivation, and strong inhibitory signals from the tumor microenvironment. We speculate that CD8^+^ T cells possess a unique mitochondrial quality control mechanism that can distinguish and accept exogenous mitochondria. Given that T cells in the tumor microenvironment are often in an energy-deficient state, we tend to believe that their mitochondrial quality control mechanism may have a highly permissive threshold regulation. In-depth studies on the transient signaling events during the mitochondrial uptake process in CD8^+^ T cells (such as Ca^2+^ flux, reactive oxygen species fluctuations, and metabolite shifts) and the posttranslational modification states of key quality control node proteins (such as PINK1, Parkin, BNIP3, and FUNDC1) will help elucidate the molecular basis of this unique tolerance. Such insights could guide strategies to trick the quality control system and improve the capacity of other cell types to accept transplanted mitochondria [15].

Furthermore, in Baldwin et al.’s [7] research, pretreating BMSCs with low-dose ethidium bromide (EtBr) severely impaired mitochondrial functional activity, yet these BMSCs still transfer dysfunctional mitochondria to recipient CD8^+^ T cells (Mito^+^ EtBr). However, the improvement in mitochondrial health observed in Mito^+^ cells was eliminated in Mito^+^ EtBr cells. These findings suggest that TNT-mediated mitochondrial transfer from BMSCs does not depend on the intrinsic activity of donor mitochondria. CD8^+^ T cells exhibit a more advanced mitochondrial quality control and a more unique response mechanism for mitochondrial autophagy compared to ECs. Additionally, in CD8^+^ T cells, regulatory circuits may restrict different modes of cell death during the formation of memory CD8^+^ T cells [16], suggesting that mitochondrial autophagy in CD8^+^ T cells may primarily be involved in regulating cell death rather than promoting mitochondrial renewal and enhancement of cell function. Given that mitochondrial transfer has been observed between BMSCs and various types of cells, the design of BMSCs as a “mitochondrial donor reservoir” is worth considering. Enhancing their mitochondrial biogenesis capability through genetic engineering (such as overexpressing PGC-1α) to achieve controllable and scalable mitochondrial supply could be another highly attractive research direction.

## Discussion and Outlook

This outstanding study reveals the potential of mitochondrial transplantation to restore T cell metabolic adaptation and function, and to enhance antitumor responses in animal models. It offers a new conceptual and technological framework for the application of organelle-based medicine in cancer immunotherapy. However, its long-term stability and clinical translatability remain to be validated. TNT-mediated mitochondrial transfer takes advantage of cell’s endogenous communication and avoids risks such as membrane damage and mitochondrial dysfunction associated with artificial transplantation. Yet, its efficiency is low (~10%) and the regulatory mechanism has not been fully elucidated. Elucidating the specific molecular mechanisms of mitochondrial transfer mediated by TLN2 (such as which motor proteins are recruited and how they interact with mitochondrial outer membrane proteins) will be a key breakthrough in improving efficiency. In the future, biomaterials or small-molecule agonists could be used to promote and stabilize TNT network formation during T cell expansion in vitro or adoptive therapy in vivo, thereby achieving an “engineered” improvement in mitochondrial transfer efficiency. This strategy of combining endogenous mechanism (TNTs) with exogenous regulation (biomaterials/molecules) may be more controllable and have more application potential than relying solely on gene editing or traditional drug intervention. In addition, using synthetic biology to engineer mitochondria, such as embedding anti-apoptotic proteins or metabolic enzymes into donor mitochondria, to endow T cells with the ability to resist acidification/hypoxia in the tumor microenvironment and develop “super donor” cells also holds broad prospects.

It will be important to explore the generalizability of mitochondrial transplantation platforms in future studies. For example, applying this technology to other immune cells prone to exhaustion or functional defects (such as regulatory T cells, natural killer cells, TILs, and dendritic cells) could empower them and broaden the scope and depth of mitochondrial transplantation in tumor immunity. In addition, T cell immunosenescence commonly arises within the TIME, influencing cancer prognosis and the efficacy of immunotherapy [17]. Senescent T cells express characteristic markers and exhibit genomic and proteostatic instability, reduced proliferative capacity, metabolic dysfunction, and epigenetic alterations, all of which lead to a marked decline in their antitumor activity [17]. In future studies, reversing or delaying T cell senescence will be key to improving the efficacy of cancer immunotherapy. This study suggests that mitochondrial supplementation can improve T cell metabolism and stress tolerance, but whether it can effectively reverse or delay T cell senescence remains unresolved. Furthermore, it is still unclear whether mitochondrial transplantation platforms can be extended to interventions for age-related diseases. We propose that future research could combine single-cell multi-omics analysis and functional assessment in elderly donors/aging models to specifically validate whether this mitochondrial transplantation platform can alleviate cell dysfunction caused by aging and broaden the scope of application of organelle medicine [18,19].
